# The clinical safety, biodistribution and internal radiation dosimetry of flutemetamol (^18^F) injection in healthy Japanese adult volunteers

**DOI:** 10.1007/s12149-015-0986-2

**Published:** 2015-06-05

**Authors:** Michio Senda, David J. Brooks, Gill Farrar, Edward J. Somer, Carolyn L. Paterson, Masahiro Sasaki, Brian J. McParland

**Affiliations:** Division of Molecular Imaging, Institute of Biomedical Research and Innovation, Kobe, Japan; GE Healthcare Life Sciences, The Grove Centre, The Grove Centre, White Lion Road, Amersham, Buckinghamshire HP7 9LL England, UK; Imperial College, London, England, UK

**Keywords:** Amyloid imaging, [^18^F]Flutemetamol, PET, Biodistribution, Dosimetry, Healthy Japanese subjects

## Abstract

**Objectives:**

The Phase I safety, biodistribution and internal radiation dosimetry study in adult healthy Japanese males of flutemetamol (^18^F) injection, an in vivo β-amyloid imaging agent, is reported and compared with previously obtained Caucasian data.

**Methods:**

Whole-body PET scans of 6 healthy volunteers (age 51.8–61.7 years) were acquired approximately 4 h post-injection (administered activity 102–160 MBq). Venous blood sampling determined ^18^F activity concentrations in whole blood and plasma and high-performance liquid chromatography (HPLC) established the percentages of parent [^18^F]flutemetamol and its metabolites. Voided urine activity was recorded. The decay-corrected and normalised ^18^F activity of 14 source organ regions as a function of time was entered into the OLINDA/EXM software to calculate the internal radiation dosimetry and effective dose of each subject following the MIRD schema. The pharmacokinetics, biodistribution and dosimetry profiles were compared to data obtained from a cohort of healthy Caucasian adult volunteers from a previous Phase I study of [^18^F]flutemetamol.

**Results:**

Flutemetamol (^18^F) injection was well tolerated. The highest mean initial uptakes were measured in the liver (15.2 %), lungs (10.2 %) and brain (6.6 %). The highest mean radiation absorbed doses were received by the gallbladder wall (366 μGy/MBq), upper large intestine (138 μGy/MBq) and small intestine (121 μGy/MBq). The mean effective dose was 34.9 μSv/MBq. HPLC analysis demonstrated that at 5-min post-injection about 75 % of plasma ^18^F radioactivity was in the form of parent [^18^F]flutemetamol, reducing to 8 and 2 % at 25 and 90 min, respectively, giving rise to less lipophilic ^18^F-labelled metabolites. Comparisons with the Caucasian cohort showed no differences that could be regarded as clinically significant.

**Conclusion:**

The clinical safety of [^18^F]flutemetamol demonstrated no differences of clinical significance in the pharmacokinetics, biodistribution and internal radiation dosimetry profiles between Caucasian and Japanese adults.

## Introduction

β-Amyloid neuritic plaques are one of the characteristic neuropathological hallmarks of Alzheimer disease (AD). The capability of the in vivo imaging of this pathology suggests the possibility to improve the accuracy of diagnosis at much earlier stages of disease and the ability to measure the future potential efficacy arising from disease-modifying therapies. Earlier imaging investigations using the ^11^C-labelled thioflavin T derivative, known as Pittsburgh compound B ([^11^C]PiB), have been previously conducted in clinically probable AD [1–3] and other dementia types [4, 5]. The short 20-min physical half-life of ^11^C restricts the use of this radionuclide (and therefore the [^11^C]PiB compound) to facilities with an on-site cyclotron where the transport time of ^11^C from the production site to the imaging site is short. A natural focus therefore has evolved towards the use of the radioisotope ^18^F, with its longer physical half-life of 110 min, in the radiolabelling of these amyloid-specific ligands.

[^18^F]Flutemetamol is a ^18^F-labelled thioflavin derivative of the Pittsburgh B compound and has been under extensive investigation of in vivo imaging of β-amyloid, mainly in Caucasians [6–8]. [^18^F]Flutemetamol is structurally identical to [^11^C]PiB apart from the inclusion of ^18^F at the 3′ position. This paper describes a Phase 1, single-centre, single-dose, open-label and non-randomised study designed to be the first to evaluate the clinical safety, biodistribution, pharmacokinetics and metabolism of ^18^F and the internal radiation dosimetry resulting from an intravenous administration of flutemetamol (^18^F) injection in a Japanese cohort of healthy adult volunteers. Clinical safety, biodistribution and internal radiation dosimetry assessments of [^18^F]flutemetamol have been performed previously with a Caucasian cohort of healthy adult volunteers [9] and these are compared with the results obtained from this work. Because ethnicity may affect transporters and metabolising enzymes that determine pharmacokinetics and biodistribution [10, 11], it is essential to confirm inter-ethnic comparability for global use of the PET drug.

## Methods and materials

Unless specified otherwise, all numerical data are provided as the mean ±1 standard deviation.

### Subjects

This study was performed at the Institute of Biomedical Research and Investigation (IBRI) in Kobe, Japan. Approval was received from the local Institutional Review Board and the study was conducted in accordance with Good Clinical Practice and the guidelines of the International Commission on Harmonisation. Six healthy adult male Japanese volunteers with a mean age of 55.3 ± 3.6 years, a whole-body weight of 72.7 ± 15.7 kg and a body-mass index of 24.4 ± 4.8 kg/m^2^ were recruited. The subject inclusion criteria included being of the Japanese race, an age exceeding 50 years, the ability to provide informed written consent, normal medical history (including physical examination, electrocardiogram, haematology and biochemistry), no evidence of cognitive impairment by medical history and a Mini-Mental State Examination score of 27 or greater.

### Radiopharmaceutical preparation

[^18^F]Flutemetamol was synthesised on a FASTlab synthesiser model by the reaction of *N*-[4-(6-ethoxymethoxy-benzothiazol-2-yl)-2-nitro-phenyl]-*N*-methylformamide (AH111907) with [^18^F]fluoride and purified by solid phase extraction followed by formulation into flutemetamol (^18^F) injection. The investigational medical product was prepared and handled according to Good Manufacturing Practice at the PET drug manufacturing site, IBRI. The manufacturing and analytical procedures, specifications and associated documentation were prepared by GE Healthcare and transferred to IBRI during a formal technology transfer process. The successful transfer was assessed by the production and analysis of a minimum of three technical batches. The measured radiochemical purity was >99.9 % for every batch used for this study based on the technical details which were defined in the testing methods for this phase 1 study.

### Administration

Flutemetamol (^18^F) injection was administered intravenously in an antecubital vein followed by a 5-ml saline flush. The mean administered activity to the six subjects was 130.2 ± 25.9 MBq (range 102.3–160.0 MBq) with an injection volume of 1.5 ± 0.56 ml (range 1.0–2.5 ml). The injected mass dose was 157 ± 54 ng (range 80–221 ng).

### Safety data collection

Safety data were collected up to 24 h after injection and included vital signs (blood pressure, respiratory rate, heart rate and body temperature); cardiovascular, lung, abdomen and neurological examinations; electrocardiogram; and laboratory parameters including serum chemistry, haematology, coagulation and urinalysis.

Venous blood samples were collected through an indwelling catheter in an antecubital vein before and 30 m, 2.5 h, 4 h and 24 h after injection of drug product.

### Pharmacokinetic measurement

Venous blood samples were taken at a nominal total of 10 time points (including a pre-injection sample) from 2 min to 4 h post-injection (pi) for measurement of ^18^F activity concentrations in whole blood and plasma. Three samples (acquired at 5, 25 and 90 min pi) were further analysed with high-performance liquid chromatography (HPLC) for the presence of the parent [^18^F]flutemetamol and ^18^F-labelled metabolites over time.

The blood samples acquired at 5, 25 and 90 min pi were collected in heparinised tubes and centrifuged at 5000 rpm, 3500 G to obtain the plasma samples. Each plasma sample was diluted 1:1 with liquid chromatography starting mobile phase [acetonitrile/water (5/95 v/v) containing 0.05 M sodium acetate (pH 5.5)] and 1.0 ml of this diluted sampled was injected directly to the column. Deproteinization was omitted to make sure the entire recovery of the plasma radioactivity to obtain accurate % parent fraction value.

Radioactive components were analysed by using HPLC system (LC-10AD, Shimadzu Co., Kyoto, Japan) with NaI(Tl) radiation detector (US-2000, Universal Giken, Odawara, Japan) to measure radioactive fractions, and the elution was monitored by UV absorbance at 330 nm. A Chromolith Performance RP-18e column (4.6 × 100 mm; Merck, Darmstadt, Germany) was used, and a flow rate of 2.0 ml/min of acetonitrile/water (5/95 v/v) containing 0.05 M sodium acetate (pH 5.5) was the initial condition used. After 1.0 min of sample injection, the ratio of acetonitrile/water was changed to 30/70 v/v for 3 min and then changed to 95/5 v/v for 16 min and maintained for the next 4 min. The columns were then washed with acetonitrile/water (5/95 v/v) containing 0.05 M sodium acetate (pH 5.5) for 4 min. A new column was used for each subject and the HPLC analytical system was primed and equilibrated at the beginning of the study day. All peaks were identified and integrated manually and the areas-under-the-curve for each peak was evaluated as a percentage of the total peak area (i.e., the total ^18^F content in circulation).

Urine was collected as voided up to the end of imaging and the ^18^F activity content in each void measured.

### Image acquisition and reconstruction

Whole-body emission images were acquired in 3D-mode on a GE Discovery ST Elite Performance PET/CT platform with a 15.7 cm axial field of view. Allowing for a 7-slice overlap, the axial field of view was 13.4 cm per bed position. Prior to each emission imaging session, a whole-body CT image was acquired for attenuation correction. The axial extent of the emission images was from the head to approximately mid-thigh in order to include the urinary bladder contents within the image. The images were composed of contiguous static positions that were acquired at a number of bed positions with this number being dependent upon the subject height.

Twelve serial three-dimensional whole-body PET images were acquired for each of the subjects beginning at 2 min pi and ending at about 4 h. The acquisition times per bed position were increased from 30 to 60 s at 90 min pi and to 120 s after 2 h pi so as to compensate for the physical decay of ^18^F.

Emission images were reconstructed using ordered-subset expectation minimisation with 8 iterations and 7 subsets with post-reconstruction smoothing using an isotropic Gaussian filter (4.29 mm full-width half-maximum). Slice thickness was 3.27 mm and pixel size was 3.91 mm^2^.

### Activity quantification

Image analysis was performed on a MIM workstation (version 6.0). Volumes-of-interest (VOI) were drawn upon the fused PET–CT data around organs and tissues that demonstrated uptake of ^18^F. The VOI included, where visible, brain, salivary glands, thyroid gland, lungs, heart, liver, gall bladder contents, spleen, intestinal tract contents, urinary bladder contents and remaining tissues. It was assumed that the ^18^F activity in the unimaged lower extremities was equal to the difference between administered activity and the sum of the imaged and excreted activities. The separate ^18^F activities in heart wall and contents were determined from the ^18^F activity measured within the VOI over the heart and then by subtracting the contribution from the heart contents estimated by the product of the measured in vitro ^18^F concentration in whole blood and the reference volume of the cardiac chamber blood [13].

The ^18^F activity data were decay-corrected to the time of injection, normalised to the administered activity and then integrated analytically as functions of time in order to yield the normalised cumulated activities (NCAs) of the source regions. The International Commission on Radiological Protection (ICRP) dynamic gastrointestinal tract model was used to calculate the NCAs of the contents of the small, upper large and lower large intestines [14]. This was done by fitting the decay-corrected and normalised activity of the total intestinal contents as a function of time to an asymptotic bi-exponential function and then using the coefficients of this fit to solve for the NCAs of the individual intestinal compartments using the set of coupled differential equations and transit times provided in [12] and [14]. A dynamic urinary bladder model was also used in order to calculate the NCA of the urinary bladder contents [15]. As recommended by the ICRP for adult subject, the urinary bladder contents’ NCA was calculated for a 3.5-h voiding interval [16].

### Internal radiation dosimetry

The internal radiation dosimetry for each subject was evaluated using the Medical Internal Radiation Dose (MIRD) schema [17] with the Organ Level Internal Dose Assessment/Exponential Modelling (OLINDA/EXM) software [18] for the Cristy–Eckerman adult male (hermaphrodite) anthropomorphic phantom [19]. From the resulting absorbed dose data for the 24 MIRD-specified target regions, the effective dose was calculated separately for each subject using the tissue weighting factors provided in ICRP Publication 60 [20]. In this calculation, the gonadal absorbed dose was taken to be the arithmetic average of the absorbed doses to the testes and ovaries, the absorbed dose to the thymus gland was used as a surrogate for the absorbed dose to the oesophagus and the absorbed dose to the colon wall was taken to be equal to the mass-weighted sum of the absorbed doses to the walls of the upper- and lower large intestines.

### Biodistribution and internal radiation dosimetry of Caucasian cohort

Although the biodistribution and internal radiation dosimetry of [^18^F]flutemetamol have been reported previously for a Caucasian cohort [9], the raw ^18^F activity data from that study were re-analysed here following the same methodologies as were used on the Japanese cohort (described above) so as to enable an appropriate comparison between the two cohorts. In particular, the ICRP gastrointestinal tract model was used on these data and the urinary bladder contents’ NCA values were evaluated for a 3.5-h voiding interval rather than the 2-h interval reported in [9]. The NCAs were evaluated analytically as described above and the organ-absorbed doses and effective dose calculated using the methods described previously.

## Results

All ^18^F activities reported are decay-corrected to the time of injection and expressed as percentages of the administered activity. Differences in NCA and dose between the two cohorts are assessed with a Student’s *t* test at a significance of *p* < 0.05.

### Safety

One treatment-emergent adverse event was reported in the form of diarrhoea during the course of the study and was deemed unrelated to the administration of flutemetamol (^18^F) injection. There was overall stability throughout the follow-up period for all parameters, including clinical laboratory variables, vital signs, and electrocardiograms. No clinically important trends or safety signals were evident.

### Biodistribution and pharmacokinetics

**Fig. 1:**
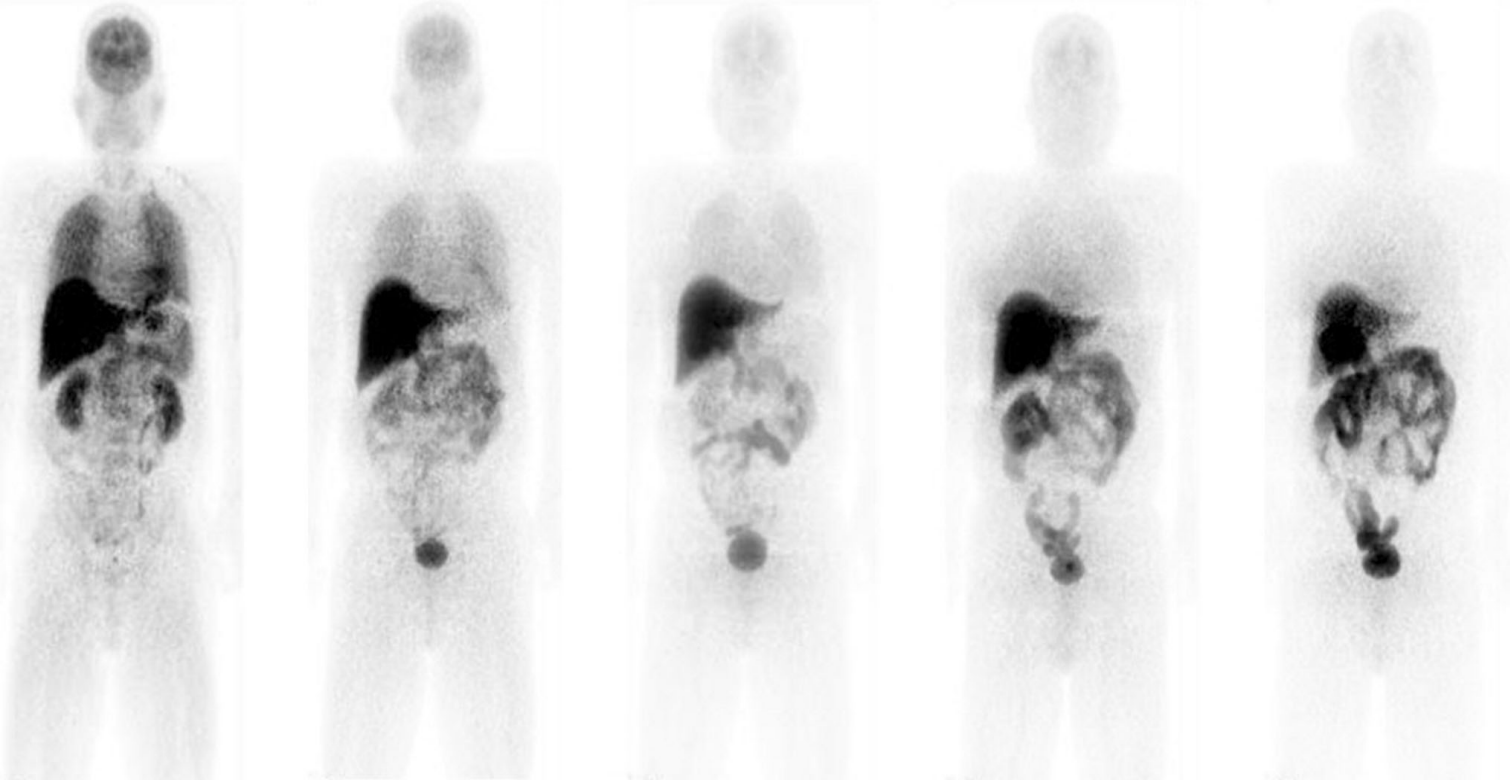
Collapsed coronal images of a representative subject following administration of flutemetamol (^18^F) injection acquired at times of (*left* to *right*) 0.10, 0.55, 1.20, 2.57 and 3.94 h post-injection

**Fig. 2:**
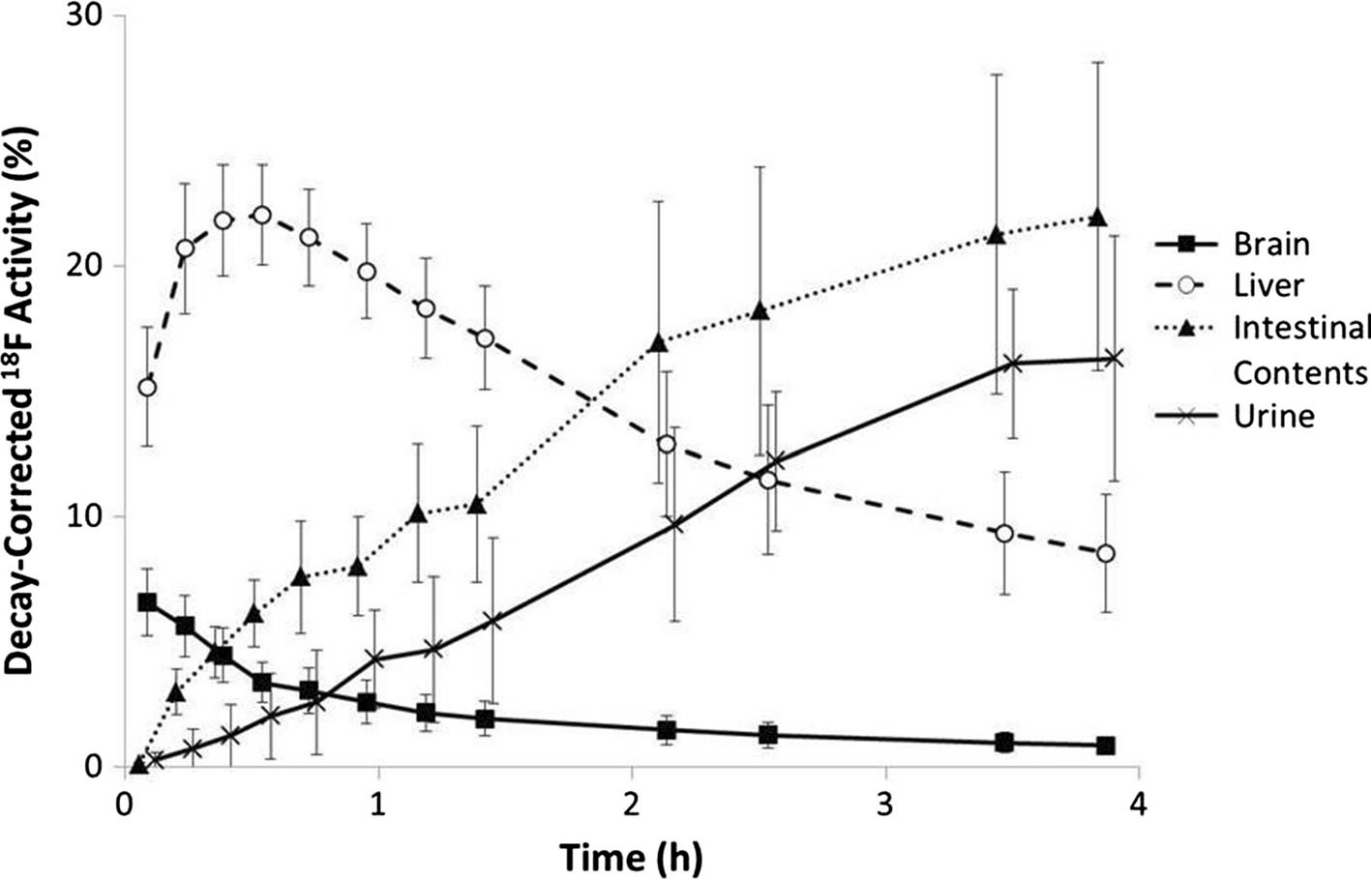
Mean decay-corrected ^18^F activity values in brain, liver, urine and intestinal contents for all six subjects as functions of time following administration of flutemetamol (^18^F) Injection. *Error bars* are drawn at ±1 standard deviation and urine and intestinal contents curves have been offset slightly for clarity

Figure 1 shows whole-body PET images of a representative subject at a number of time points following the administration of flutemetamol (^18^F) injection. Figure 2 shows the mean time–activity curves for liver, brain, urine and intestinal contents.

Excluding the remaining tissues category, the three source regions with the highest mean initial ^18^F activity uptake (defined as that measured at the initial imaging time point) were liver at 15.2 % (range 13.4–19.5 %), lungs at 10.2 % (range 5.6–12.8 %) and brain at 6.6 % (range 4.4–8.3 %). Washout of ^18^F activity from brain was generally rapid and activity was reduced to half of its initial uptake by about 1 h pi. Uptake in the liver rose to a mean of 22 % approximately 50 min pi before falling to around half this value 2 h later. Excretion of ^18^F activity was distributed between the renal pathway with 32.6 ± 10.5 % and the hepatobiliary pathway with 31.9 ± 16.9 %. Table 1 summarises the NCA values determined for the Japanese cohort of this work and for the Caucasian cohort of Ref. [9].

**Table 1:** Normalised cumulated activities of flutemetamol (^18^F) injection

Organ/tissue	Normalised cumulated activities (MBq h/MBq)			
	Japanese (this work)		Caucasian^a^	
	Mean	Standard deviation	Mean	Standard deviation
Brain	0.059	0.016	0.061	0.006
Lungs	0.102	0.030	0.063	0.008
Heart				
Wall	0.012	0.010	0.009	0.003
Contents	0.015	0.003	0.016	0.004
Spleen	0.009	0.010	0.008	0.003
Liver	0.367	0.048	0.399	0.051
Gall bladder contents	0.218	0.048	0.169	0.158
Intestinal contents				
Small intestine	0.505	0.293	0.429	0.155
Upper large intestine	0.277	0.161	0.236	0.085
Lower large intestine	0.051	0.029	0.042	0.015
Kidneys	0.052	0.023	0.029	0.020
Urinary bladder wall (3.5-h voiding interval)	0.224	0.058	0.287	0.105
Remaining tissues	1.151	0.113	0.817	0.132

^a^Calculated from the data of [9]

**Fig. 3:**
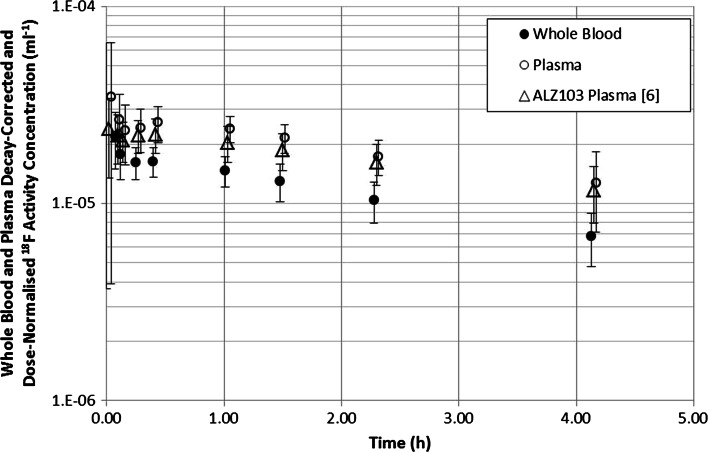
Decay-corrected ^18^F activity concentration in whole blood and plasma for all six subjects as a function of time following administration of flutemetamol (^18^F) injection. Data points, offset for clarity, are mean values and uncertainty *error bars* are ±1 standard deviation. Plasma data from a previous Caucasian study (ALZ103) [6] are also shown for comparison

**Fig. 4:**
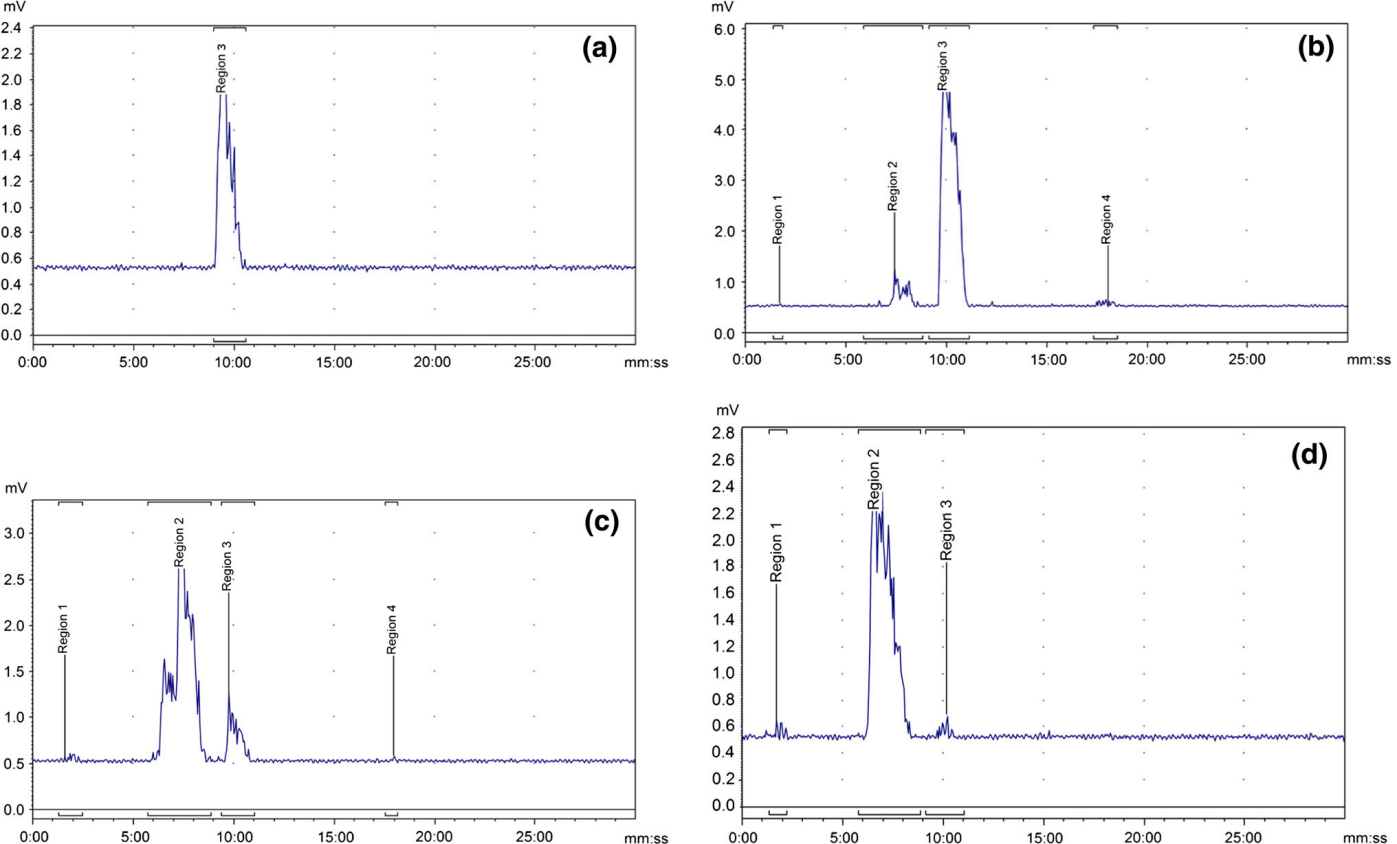
HPLC radiochromatograms for **a** plasma sample spiked ex vivo with standard [^18^F]flutemetamol, and for the plasma obtained from a representative subject at **b** 5, **c** 30 and **d** 90 min after administration of flutemetamol (^18^F) injection. The *vertical axis* shows “Radioactivity detector response (mV)” while the “Retention time (min)” appears on the horizontal axis. Region 3 corresponds to the [^18^F]flutemetamol parent in **b**, **c** and **d** as identified in **a**. Regions 1 and 2 correspond to metabolites that are more hydrophilic than the parent of Region 3, and Region 4 represents a minor lipophilic metabolite

Figure 3 presents the mean washout of activity from whole blood and plasma. The plasma activity data from the previous study on Caucasians (ALZ103) [6] are also shown for comparison in Fig. 3. Figure 4 shows the HPLC traces of ^18^F activity in plasma from a representative subject. The signals of hydrophilic species exhibit a decreased retention time. Figure 4a shows the radiochromatogram for a human plasma sample spiked ex vivo with the parent [^18^F]flutemetamol and Fig. 4b–d shows radiochromatograms obtained at 5, 25 and 90 min pi, respectively. When the total radioactivity recovered in the HPLC effluent was compared with the activity in each plasma sample applied to the HPLC, the recovery rate (effluent/plasma) was 0.98 ± 0.16, indicating that all the activity in the plasma samples was accounted for in the radio-HPLC analysis.

**Fig. 5:**
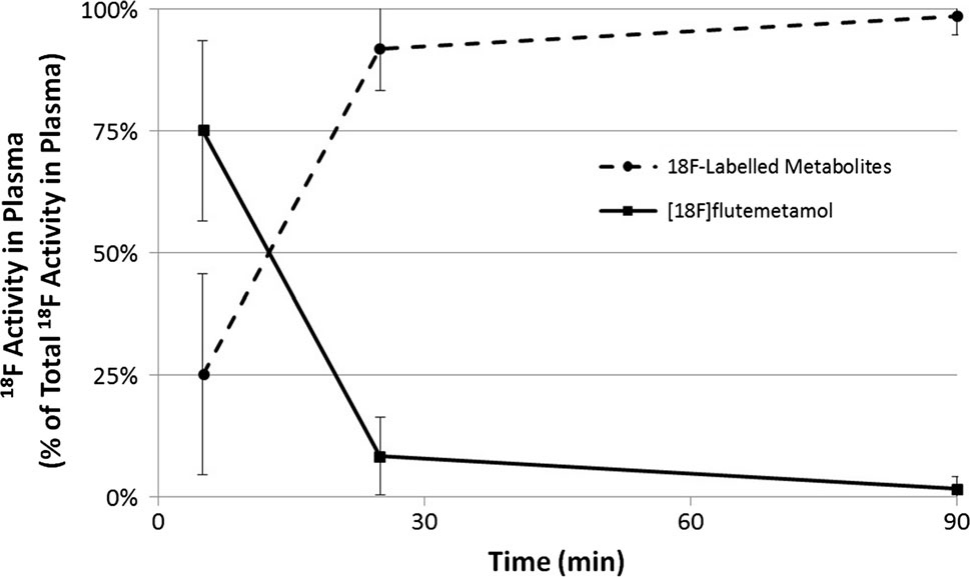
Mean areas-under-the-curve for radiochromatograms for [^18^F]flutemetamol parent and ^18^F-labelled metabolities in plasma as function of time following administration of flutemetamol (^18^F) injection. *Error bars* are ±1 standard deviation. The fraction of the parent compound ([^18^F]flutemetamol) in the plasma decreased from 75 % at 5 min to 8.3 % at 25 min with the majority of the plasma activity bound to hydrophilic metabolites

Figure 5 presents the mean ^18^F content in the form of the [^18^F]flutemetamol parent and ^18^F-labelled metabolites as a function of time following the administration of flutemetamol (^18^F) injection. A large fraction of the radioactivity was in the form of the parent [^18^F]flutemetamol at 5 min pi, which was rapidly metabolised into hydrophilic compounds at the later phases.

### Internal radiation dosimetry

The absorbed doses per unit administered activity to the MIRD-specified organs for the Japanese and Caucasian cohorts are provided in Table 2.

**Table 2:** Organ and tissue-absorbed doses normalised to the administered activity for flutemetamol (^18^F) injection

Organ/tissue	Absorbed dose per unit administered activity (mGy/MBq)			
	Japanese (this work)		Caucasian^a^	
	Mean	Standard deviation	Mean	Standard deviation
Adrenal glands	1.51E−02	8.29E−04	1.29E−02	1.64E−03
Brain	1.11E−02	2.74E−03	1.13E−02	9.01E−04
Breasts	6.70E−03	3.28E−04	5.19E−03	6.17E−04
Gall bladder wall	3.66E−01	7.22E−02	2.87E−01	2.46E−01
Stomach	1.51E−02	2.55E−03	1.23E−02	1.00E−03
Intestinal walls				
Small	1.21E−01	6.28E−02	1.02E−01	3.21E−02
Upper large	1.38E−01	7.14E−02	1.17E−01	3.60E−02
Lower large	4.90E−02	2.25E−02	4.17E−02	1.23E−02
Heart wall	1.73E−02	5.73E−03	1.45E−02	2.84E−03
Kidneys	4.42E−02	1.46E−02	3.12E−02	8.61E−03
Liver	5.55E−02	5.53E−03	5.72E−02	8.20E−03
Lungs	2.31E−02	5.01E−03	1.57E−02	1.53E−03
Muscle	1.05E−02	1.23E−03	8.69E−03	7.06E−04
Ovaries	2.86E−02	1.04E−02	2.49E−02	5.84E−03
Pancreas	1.81E−02	1.33E−03	1.50E−02	2.75E−03
Red marrow	1.18E−02	2.05E−03	1.34E−02	8.46E−04
Osteogenic cells	1.20E−02	9.22E−04	1.14E−02	8.17E−04
Skin	6.38E−03	4.55E−04	5.07E−03	4.14E−04
Spleen	1.75E−02	1.00E−02	1.46E−02	3.29E−03
Testes	8.64E−03	5.63E−04	7.52E−03	8.60E−04
Thymus gland	7.77E−03	3.93E−04	5.97E−03	7.46E−04
Thyroid gland	6.49E−03	5.55E−04	6.38E−03	1.56E−03
Urinary bladder wall	1.17E−01	2.75E−02	1.45E−01	4.99E−02
Uterus	2.74E−02	7.64E−03	2.54E−02	5.89E−03
ED (mSv/MBq)	3.49E−02	1.04E−02	3.24E−02	6.48E−03

^a^Calculated using the NCA data of Table 1

In this work for the Japanese cohort, the three organs with the largest absorbed doses were the gallbladder wall (366 ± 72.2 μGy/MBq), upper large intestine (138 ± 71.4 μGy/MBq) and the small intestine (121 ±62.8 μGy/MBq). The effective dose was calculated to be 34.9 ± 10.4 µSv/MBq.

For the Caucasian cohort, the three organs with the largest absorbed doses were the gall bladder wall (287 ± 24.6 μGy/MBq), urinary bladder wall (145 ± 49.9 μGy/MBq) and the wall of the upper large intestine (117 ± 36.0 μGy/MBq). The effective dose was calculated to be 32.4 ± 6.5 µSv/MBq.

## Discussion

### Safety

The design of this study was to evaluate the biodistribution of ^18^F and the internal radiation dosimetry associated with [^18^F]flutemetamol in healthy adult Japanese volunteers as well as the clinical safety of the drug product flutemetamol (^18^F) injection. Flutemetamol (^18^F) injection was found to be safe and well tolerated with one adverse event having occurred in six subjects. This event was mild and was judged to not be associated with the radiopharmaceutical. Excretion of ^18^F in both the Japanese and Caucasian cohorts was predominantly renal and the highest absorbed doses were received by the urinary bladder wall, kidneys and the liver.

### Pharmacokinetics and metabolism

This study demonstrates that the plasma time–activity curve is similar to the Caucasian data acquired in the previous study [6] (Fig. 3). HPLC analysis demonstrates that [^18^F]flutemetamol is rapidly metabolised with only 8.3 ± 8 % present as the parent in circulation at 25 min pi (Fig. 5). By that time 89.7 ± 8.5 % of ^18^F in the circulation is represented by a metabolised species (region 2 in Fig. 4b–d) more hydrophilic than the parent. These results are not much different from the Caucasian data in [6] at least during the initial 20–25 min pi, in which the fraction of intact [^18^F]flutemetamol was 84.7 % (normal) and 77.5 % (AD) at 2 min but was decreased to 23.8 % (normal) and 27.0 % (AD) at 20 min and the activity turned into hydrophilic metabolites. The parent fraction in the later phase was lower than the Caucasian data, which is difficult to interpret due to poor count statistics and the difference in the equipment. With hindsight, the sensitivity of radio-detector used in the HPLC analysis in the present study was not sufficient for high-precision metabolite analysis. As a result the tube volume was increased, resulting in wide peaks shown in the charts of Fig. 4, which might have masked small peaks close to the parent compound. Within such methodological limitation of the study, ethnic difference in pharmacokinetics and metabolism was minimal.

A small peak representing a lipophilic labelled metabolite was detected in the HPLC analysis, see “Region 4” in Fig. 4b, c. Although a lipophilic compound may enter the brain, this would not influence the brain uptake measured with PET because the amount is small.

Because the plasma activity concentration and the fraction of intact [^18^F]flutemetamol constitute the input function to the brain, their similarity supports the fundamentals for the usefulness of brain PET imaging with flutemetamol (^18^F) injection in the Japanese population to the same extent as in the Caucasian population.

### Comparison of biodistribution and internal radiation dosimetry with Caucasian population

An earlier investigation of the biodistribution and internal radiation dosimetry of flutemetamol (^18^F) injection in a Caucasian population was described in [9]. Tables 1 and 2 include our re-evaluations of the NCAs, organ-absorbed doses and effective dose for that population. The dosimetry data for the Caucasians presented here differ slightly from those given by [9]. This is a consequence of having derived the Caucasian results in the same manner as those of the Japanese results using the procedures described in this paper. In particular, the NCA for the urinary bladder contents was calculated for a 2-h voiding interval in Ref. [9], whereas in this paper the ICRP-recommended 3.5-h voiding interval was used. In Table 1, we see no significant difference between the NCAs calculated for source regions in the Japanese and Caucasian cohorts, with the exceptions of the lungs and the remainder category. However, these differences do not cause any issues from the standpoint of clinical radiation exposure.

The organ-absorbed doses between the Japanese and Caucasian cohorts are largely in agreement and, when ranked in order of magnitude, it is only with the lungs in tenth position that we begin to see any significant difference in absorbed dose. There is no statistical difference in effective doses between the two the cohorts.

## Conclusions

The first safety, biodistribution and internal radiation dosimetry profiles in a Japanese healthy adult population following the administration of flutemetamol (^18^F) injection have been presented. It was demonstrated that flutemetamol (^18^F) injection is safe and has an acceptable radiation dosimetry profile. The radiopharmaceutical is rapidly metabolised to form a species more hydrophilic than the [^18^F]flutemetamol parent. A comparison of these data with those obtained before for a Caucasian population has demonstrated no differences of clinical significance between these two populations for flutemetamol (^18^F) Injection.
